# Adverse body composition and lipid parameters in patients with prolactinoma: a case-control study

**DOI:** 10.1186/s12902-021-00733-6

**Published:** 2021-04-26

**Authors:** Anna Sophia Posawetz, Christian Trummer, Marlene Pandis, Felix Aberer, Thomas R. Pieber, Barbara Obermayer-Pietsch, Stefan Pilz, Verena Theiler-Schwetz

**Affiliations:** Division of Endocrinology and Diabetology, Department of Internal Medicine, Medical University of Graz, Auenbruggerplatz 15, 8036 Graz, Austria

**Keywords:** Hyperprolactinaemia, lipid metabolism, total cholesterol, LDL cholesterol, fat mass, prolactin

## Abstract

**Background:**

Hyperprolactinaemia might cause adverse metabolic effects. The aim of our study was to compare parameters of body composition, glucose and lipid metabolism between untreated patients with prolactinoma and controls and to assess changes after initiation of cabergoline.

**Methods:**

Case-control study with a retrospectively analyzed follow-up in patients with prolactinoma after initiation of cabergoline therapy.

**Results:**

21 patients with prolactinoma (9 micro- and 12 macroprolactinomas; 7 females) and 30 controls were analyzed. Patients with prolactinoma had significantly higher BMI than controls; fat mass did not differ between groups. Only men - but not women - with prolactinoma had significantly higher fat mass at all six sites measured compared to controls. Levels of LDL (130 (107–147.5) vs. 94.5 (80–127.5) mg/dl, *p* < 0.001) were significantly higher, levels of HDL (56 ± 16.7 vs. 69.2 ± 14.6 mg/dl, *p* = 0.004) significantly lower than in controls. Fasting glucose, HOMA-IR, HbA1c, adiponectin, CRP, and homocysteine did not differ between groups.

After a median of 10 weeks (IQR 7–18 weeks) after initiation of cabergoline, total (from 212.5 ± 36.2 to 196.9 ± 40.6 mg/dl, *p* = 0.018) and LDL cholesterol (130 (107–147.5) to 106.5 (94.3–148) mg/dl, *p =* 0.018) had significantly decreased. Analyzing men and women separately, this change occurred in men only.

**Conclusions:**

Reasons for the association between prolactin and metabolic parameters include direct effects of prolactin on adipose tissue, hyperprolactinaemia-triggered hypogonadism and dopamine-agonist therapy per se. Altered lipid metabolism in patients with prolactinoma might imply an increased cardiovascular risk, highlighting the necessity to monitor metabolic parameters in these patients.

## Introduction

In addition to effects on gonadal function, hyperprolactinaemia caused by prolactin secreting tumors of the pituitary gland or the intake of antipsychotic drugs might have effects on food intake, weight gain as well as on glucose and lipid metabolism.

Weight gain, increased body fat content in untreated patients with prolactinoma as shown in some studies [1] and reduction of waist circumference after cabergoline treatment [2] might be linked to the influence of prolactin on appetite-regulating systems [3–5]. In contrast, other studies failed to show a difference in body fat in women as compared to controls [6] or a change in body mass index (BMI) after 6 months of cabergoline treatment in women [6] or both men and women [7].

Likewise, current data on glucose metabolism in patients with prolactinoma are inconsistent. Patients with untreated prolactinomas were shown to have higher serum insulin and homeostasis model assessment insulin resistance (HOMA-IR), as well as lower adiponectin levels [8] and impaired glucose tolerance [9] compared to controls. Treatment with cabergoline was followed by a decrease in insulin after 12 and 60 months [10], HOMA-IR after six to 60 months [2, 7, 10], Glycated haemoglobin A1c (HbA1c) after 12 months of cabergoline [2], and fasting glucose levels after 6 months [7]. Measures of insulin sensitivity increased after six and 12 months of cabergoline treatment [2, 11]. In our previously published, retrospective study including 53 patients with prolactinomas, however, no effect on fasting glucose and HbA1c was seen after a median of nine months of cabergoline treatment [12].

Concerning lipid metabolism, we did, however, observe a change in low-density lipoprotein (LDL) and total cholesterol, findings that are well supported by other studies showing reductions in LDL [2, 7, 11] and total cholesterol [2] over six to 12 months of cabergoline treatment. Further, there is evidence of decreasing triglyceride [2, 7] and increasing high-density lipoprotein (HDL) levels [2]. Furthermore, carotid-intima-media-thickness decreased significantly after 6 months of cabergoline treatment, as did C-reactive protein and homocysteine [11]. Pathophysiological reasons for the association between prolactin and metabolic parameters include a possible direct effect of prolactin on adipose tissue, hyperprolactinaemia-triggered hypogonadism and dopamine-agonist therapy per se.

Due to the inconsistency of the published data, the aim of the present study was to compare parameters of body composition, glucose and lipid metabolism between untreated patients with prolactinoma and controls. Further, we aimed to assess changes in glucose and lipid parameters in patients with prolactinomas at baseline and at first follow-up after initiation of cabergoline. We hypothesized that compared to controls untreated patients with prolactinoma would show impaired glucose and lipid parameters, which would ameliorate after treatment with cabergoline.

## Subjects and methods

All methods were carried out in accordance with current guidelines and regulations.

### Subjects

For this case-control study, patients with newly diagnosed and untreated micro- and macroprolactinoms presenting in the outpatient clinic of the Division of Endocrinology and Diabetology, Department of Internal Medicine, Medical University of Graz, Austria, between September 2014 and May 2019 were screened for eligibility and if suitable asked for participation in the study. Inclusion criteria were men and pre- and postmenopausal women with the diagnosis of micro- or macroprolactinoma according to the guidelines of the Endocrine Society [13]. These criteria include clinical signs of hyperprolactinaemia, elevated serum prolactin levels, and a pituitary tumor on magnetic resonance imaging (MRI). As serum prolactin levels generally parallel tumour size in patients with prolactinomas, macroprolactinomas are typically associated with prolactin levels greater than 250 ng/ml. In cases of lower prolactin levels in combination with a pituitary macroadenoma on MRI, pituitary stalk compression was considered and ruled out as the main differential diagnosis [13].

Patients with secondary hyperprolactinaemia due to drugs (including neuroleptics, antidepressants, opiates and gastrointestinal prokinetics), patients with mixed-secreting tumours and patients already on dopamine agonists were excluded. Further, patients receiving oestrogens and/or progesterone as contraceptives, postmenopausal women on hormone replacement therapy and patients on antidiabetic therapy or on medications influencing lipid metabolism (statins, fibrates, ezetimibe, proprotein convertase subtilisin/kexin type 9 (PCSK9) inhibitors) were excluded. Additionally, patients with multiple pituitary hormone deficiencies and patients with hypogonadism due to causes other than prolactinoma itself were not eligible for inclusion into the study.

For the control group, healthy volunteers were recruited in the outpatient clinic of the Division of Endocrinology and Diabetology. Exclusion criteria included: antidiabetic treatment, lipid-lowering therapy, intake of oral contraceptives or hormone replacement therapy, pituitary or adrenal diseases, hypo- or hyperthyroidism, coronary artery disease or peripheral artery disease. The Ethics Committee of the Medical University of Graz approved this study.

The follow-up of prolactinoma patients was planned retrospectively. Again, the Ethics Committee of the Medical University of Graz approved the study. All study participants gave written informed consent before carrying out any study related procedures.

#### Procedures

Standard anthropometric data (height, weight, waist and hip circumference, blood pressure) were obtained from patients and controls. BMI was calculated as the weight in kilograms divided by the square of height in meters. Basal blood samples were obtained between 8.00 and 9.00 after an overnight fast for determination of hormonal (prolactin, total testosterone (TT), free testosterone (FT), free triiodothyronine (fT3), free thyroxine (fT4), thyroid-stimulating hormone (TSH), cortisol, adrenocorticotropic hormone (ACTH), human growth hormone (HGH), insulin-like growth factor 1 (IGF-1) and metabolic parameters (glucose, insulin, total, LDL, HDL cholesterol, triglycerides). In the first step, patients and controls were compared. Secondly, hormonal and metabolic parameters in patients with prolactinomas were compared at baseline and first follow-up.

#### Biochemical analyses

Prolactin, total testosterone, TSH, fT3, fT4, cortisol, ACTH, were measured by luminescence immunoassay (Siemens, Erlangen, Germany), insulin was determined by ELISA (Siemens, Erlangen, Germany). Modular Analytics SWA (Roche, Basel, Switzerland) were used for measuring fasting glucose, HbA1c, triglycerides, total cholesterol, HDL cholesterol, and LDL cholesterol. Coefficients of variation for all parameters analyzed were < 10%. Adiponectin was measured by radioimmunoassay (Millipore).

#### Dual energy X-ray absorptiometry

Fat mass was measured by dual energy X-ray absorptiometry (Lunar iDXA, GE Healthcare). The following sites were analyzed: arms, legs, trunk, android, gynoid, and total body.

### Statistical analyses

Data are presented as mean and standard deviation (SD) when normally distributed or median and interquartile ranges (IQR) when not normally distributed. Distribution of data was determined by Kolmogorov-Smirnov test and descriptive statistics. All continuous parameters following a non-normal distribution were logarithmically transformed when parametric tests were applied.

For comparisons between groups the student’ T-Test or the Mann-Whitney-test was used, for comparisons between baseline and follow-up within the prolactinoma group, the paired student’s T-Test or the Wilcoxon test was applied. ANOVA was used for comparisons between groups with adjustment for BMI. In terms of sex hormones, men and women were analysed separately to account for the different reference ranges of oestradiol and testosterone in males and females, postmenopausal women were excluded from analyses. A *p*-value of 0.05 was considered statistically significant. All analyses were performed using SPSS 25.0 (SPSS Inc., Chicago, IL).

## Results

Twenty-seven patients were enrolled in the study in total, of which six were excluded (three due to a screening failure, two due to statin therapy, one patient due to a previously started cabergoline therapy). Twenty-one patients with prolactinoma thus remained for analysis. Thirty-one healthy controls were recruited, one had to be excluded due to mild hyperprolactinaemia and therefore unclear diagnosis of prolactinoma. Baseline characteristics are shown in Table 1.

**Table 1:** A: Baseline characteristics in patients with prolactinoma and controls; B: Metabolic and endocrine parameters in patients with prolactinoma at follow-up

A	Controls	IQR or SD	Prolactinomas	IQR or SD	***p***-value controls vs. prolactinomas	B	IQR or SD	***p***-value baseline vs. follow-up
						Prolactinomas follow-up		
	Median or mean		Median or mean			Median or mean		
**Sex**	18 female (3 post-, 15 premenopausal), 12 male		7 female (all premenopausal), 14 male					
**Age (years)**	36.7	11.0	40.0	17	0.418			
**BMI (kg/m**^**2**^**)**	22.9	21.0–26.6	27.5	22.4–33.5	0.018	27.5	21.5–35.0	0.686
**BMI (kg/m**^**2**^**) in categories in absolute numbers (percent)**								
**−18.4 (underweight)**	1 (3.3%)		1 (4.8%)			0 (0%)		
**18.5–24.9 (normal)**	20 (66.7%)		6 (28.6%)			2 (9.5%)		
**25–29.9 (overweight)**	7 (23.3%)		7 (33.3%)			2 (9.5%)		
**30–34.9 (obese class I)**	1 (3.3%)		4 (19%)			1 (4.8%)		
**35–39.9 (class II)**	0 (0%)		3 (14.3%)			1 (4.8%		
**≥ 40 (class III)**	1 (3.3%)		0 (0%)			0 (0%) 15 (71.4%) missing		
**WHR**	0.86	0.06	0.89	0.12	0.309			
**Systolic BP (mmHg)**	129	17	129	21	0.997			
**Diastolic BP (mmHG)**	85	13	84	15	0.921			
**Prolactin (ng/ml)**	9.3	7.5–12.4	247.7	105.0–722.6	< 0.001	11.6	7.6–30.4	< 0.001
**Total cholesterol (mg/dl)**	192.6	45.0	212.5	36.2	0.099	196.9	40.6	0.018
**LDL (mg/dl)**	94.5	80–127.5	130	107–147.5	< 0.001	106.5	94.3–148	0.018
**HDL (mg/dl)**	69.2	14.6	56.0	16.7	0.004	55.7	21.2	0.543
**Triglycerides (mg/dl)**	66.5	53.8–101.0	84.0	62.0–159.0	0.075	84.5	60–168.3	0.142
**Fasting glucose (mg/dl)**	89	86–93	88	86–96	0.939	87	82–91.5	0.191
**HOMA-IR**	1.89	1.40–3.11	2.32	1.64–3.59	0.151			
**HbA1c (mmol/mol)**	33	31.0–34.0	34	32.5–35.5	0.069			
**Adiponectin (μg/ml)**	8.8	6.1–13.3	9.3	7.0–11.5	0.723			
**Homocystein (μmol/l)**	9.6	8.0–11.2	8.8	8.0–12.3	0.926			
**CRP (mg/l)**	0.8	0.6–2.0	1.3	0.6–3.9	0.427			
**Estradiol (premenopausal women only) (pg/ml)**	162.5	69.4	63.0	47.0	0.030	61.8	39.2	0.318
**Total testosterone (men only) (ng/ml)**	3.8	1.5	1.3	0.6	< 0.001	2.4	1.0	< 0.001
**Free testosterone** **(men only) (ng/dl)**	16.6	4.6	5.6	2.7	< 0.001	9.6	4.2	0.006
**LH (mIE/ml)**	6.3	3.9–10.3	3.6	1.7–5.1	0.001	4.0	2.6–5.2	0.171
**FSH (mIE/ml)**	5.8	3.9–7.9	3.4	2.5–6.1	0.003	4.7	3.3–5.5	0.020
**TSH (**μU**/ml)**	2.1	0.8	1.9	0.7	0.421	2.3	1.2	0.333
**fT4 (pmol/L)**	15.0	13.3–16-0	13.4	12.5–14.7	0.051	14.9	12.4–15.9	0.127
**fT3 (pmol/L)**	4.9	0.7	4.1	0.7	< 0.001	4.7	0.6	< 0.001
**% fat arms**	30.5	9.9	31.7	7.7	0.619			
**% fat legs**	30.4	9.4	31.3	6.9	0.716			
**% fat trunk**	31.7	10.2	36.8	11.1	0.099			
**% fat android**	32.1	12.4	38.5	13.7	0.089			
**% fat gynoid**	33.6	9.7	35.5	7.6	0.463			
**% fat total body**	30.7	8.6	33.3	8.0	0.282			

Patient characteristics and laboratory parameters at baseline in patients and controls and at follow-up in patients. Data are presented as mean and standard deviation or median and interquartile range depending on distribution of data.

*BMI* body mass index, *WHR* waist-hip ratio, *LDL* low-density lipoprotein, *HDL* high-density lipoprotein, *HOMA-IR* homeostatic model assessment of insulin resistance, *HbA1c* glycated haemoglobin, *CRP* C-reactive protein, *LH* luteinizing hormone, *FSH* follicle-stimulating hormone, *TSH* thyroid-stimulating hormone, *fT4* free thyroxine, *fT3* free triiodothyronine;

Of the 21 patients with prolactinoma, nine had micro- and 12 had macroprolatinoma. By definition, patients had significantly higher levels of prolactin and lower levels of luteinizing hormone (LH) and follicle-stimulating hormone (FSH). Women with prolactinoma – all premenopausal in this study – had lower levels of estradiol, men with prolactinoma lower levels of total and free testosterone (see Table 1). 5/9 patients with microprolactinoma and 11/12 with macroprolactinoma had hypogonadism. At baseline, only one patient had a fasting glucose ≥126 mg/dl and an elevated HbA1c of 67 mmol/mol, thus previously untreated diabetes mellitus. No patient had impaired fasting glucose (defined as fasting glucose from 110 to 125 mg/dl). After exclusion of the patient with previously untreated diabetes mellitus, results between groups and in patients with prolactinomas before and after treatment remained materially unchanged (data not shown).

**Fig. 1:**
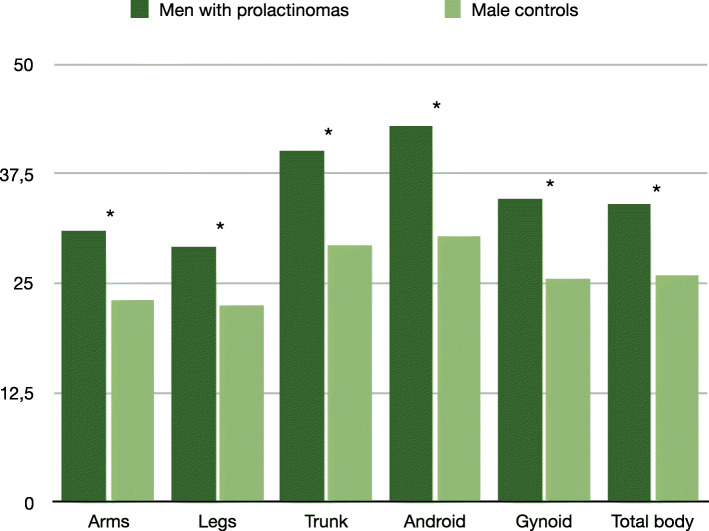
Fat distribution (in %) in men with untreated prolactinoma versus controls. * = significant

Of the 30 patients in the control group, 18 were female, 12 were male, with a mean age of 36.7 ± 11. Patients with prolactinoma had significantly higher BMI than controls, but were otherwise comparable to the control group. Concerning body composition, fat mass measured by dual-energy X-ray absorptiometry (DXA) at six sites (arms, legs, trunk, android, gynoid, total body) did not differ between groups when analyzing all patients together (Table 1). Analyzing men separately, we observed a significantly higher fat mass at all six sites in patients as compared to controls (arms: 31 ± 6.2 vs. 23.0 ± 7.1%, *p* = 0.006; legs: 29.2 ± 5.8 vs. 22.4 ± 5.6%, *p =* 0.003; trunk: 40.1 ± 8.0 vs. 29.3 ± 10.1%, *p =* 0.009; android: 43.0 ± 9.0 vs. 30.4 ± 12.8%, *p* = 0.006; gynoid: 34.7 ± 6.5 vs. 25.5 ± 7.2%, *p* = 0.003; total body: 34.0 ± 6.9 vs. 25.9 ± 7.4%, *p* = 0.020; Fig. 1). In women, no difference in body fat mass was observed between patients and controls (data not shown).

**Fig. 2:**
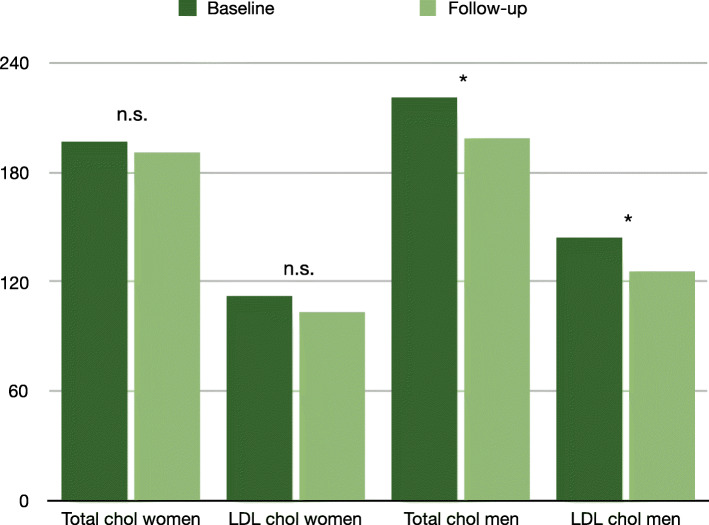
Change in total and LDL cholesterol (in mg/dl) in women and men. * = significant. n.s. = not significant. LDL = low-density lipoprotein. Chol = cholesterol

Total cholesterol and triglycerides did not differ between groups, but levels of LDL were significantly higher and levels of HDL were significantly lower than in controls (Fig. 2). Fasting glucose, HOMA-IR, HbA1c, adiponectin, C-reactive protein (CRP), homocysteine did not differ between groups. After adjustment for BMI (univariate ANOVA), levels of LDL and HDL cholesterol remained significantly different between groups (*p* = 0.015 and 0.0.17, respectively).

Sixteen patients received a standard dose of cabergoline of 0.25 mg twice per week, five patients received a deviating dose due to extremely high or very low levels of prolactin (the minimum dose of cabergoline was 0.25 mg per week, the maximum dose was 1.5 mg per week). After a median routine follow-up of 10 weeks (IQR 7–18 weeks), all patients with prolactinoma had significantly decreased levels of prolactin. In four patients prolactin was still slightly above the upper reference range of 17.7 ng/mL, in three patients the elevation was still higher, partly because two of these patients had had very high levels of prolactin at baseline. Total and LDL cholesterol had significantly decreased, while HDL, triglycerides and fasting glucose had not changed (see Table 1). Analyzing men (*n* = 13) and women (*n* = 5) separately, there was no significant decline in total and LDL cholesterol in women (total cholesterol decreased from 197.2 ± 20.0 to 191.2 ± 39.8 mg/dl, *p* = 0.720, LDL cholesterol from 112.6 ± 18.7 to 103.8 ± 27.1 mg/dl, *p* = 0.596), but indeed in men (total cholesterol decreased from 221.2 ± 42.1 to 199.1 ± 42.3 mg/dl, *p* = 0.009, LDL cholesterol from 144.6 ± 37.1 to 125.6 ± 37.5 mg/dl, *p* = 0.008). Analysing patients with micro- and macroprolactinomas separately, the decrease in total cholesterol and LDL remained significant in the group with macroprolactinomas, but not in the group with microprolactinomas (data not shown).

Total and free testosterone (in men) and FSH (in all patients), but not estradiol (in women) and LH (in all patients) already showed a significant increase after a median of 10 [7–18] weeks of cabergoline therapy (Table 1). Due to the retrospective character of the follow-up analysis, BMI at follow-up was only available in six patients. In these patients, however, no significant change could be observed (Table 1).

## Discussion

In this case control study, men and women with prolactinoma had higher BMI than controls, but only men with prolactinoma also had higher body fat content compared to healthy men. In line with most published data, we found evidence of impaired lipid metabolism in untreated patients with prolactinoma and an amelioration after initiation of cabergoline therapy. Concerning glucose metabolism, we found no alteration in patients with prolactinoma, which parallels our previously published data from a retrospective prolactinoma cohort [12].

Our findings of an elevated BMI in men and women and higher body fat in men with prolactinoma compared to controls in our study supports previously published data [1] and might be explained by the increases in food intake and weight gain due to chronic hyperprolactinaemia, as shown in animal models [14, 15]. Elevated levels of prolactin seem to cause a functional blockade of dopaminergic tone. As dopaminergic tone increases energy expenditure and causes a reduction in food intake, a blockade would promote weight gain [3, 5, 16–18].

Further, prolactin has been shown to directly stimulate the development of adipose tissue, as shown in a model of prolactin-deficient mice [19] and in studies in humans [17, 18]. This direct effect on adipose tissue in humans might be exerted via four prolactin receptor isoforms found in human adipose tissue [20]. Further, prolactin might suppress lipid storage as well as the release of adipokines such as adiponectin, interleukin-6 and possibly leptin [3]. Additionally, hyperprolactinaemia-induced hypogonadism could contribute to an adverse body composition especially in men, as androgen deficiency itself increases body fat and facilitates lean mass reduction. This has been shown in men with prostate cancer on androgen deprivation therapy [21] and by studies of testosterone replacement enhancing skeletal muscle [22], decreasing fat mass and increasing lean mass [23]. In untreated men with prolactinoma [1], higher fat percentage in the arms and the total body has been previously observed, whereas our data even show higher body fat at all six sites investigated.

In consistency with previously published data from cross-sectional studies, our data strongly support the assumption that hyperprolactinaemia might lead to impaired lipid metabolism [2, 7, 11]. In detail, hyperprolactinaemia might cause elevated levels of at least LDL cholesterol and possibly total cholesterol, a condition that can be ameliorated by adequate treatment with cabergoline, as likewise shown by Auriemma [10]. In this study, the authors were able to show that total cholesterol and in trend also LDL cholesterol had normalized after 12 months of cabergoline treatment. After 60 months triglycerides, HDL, weight and BMI as well as visceral adiposity index had improved. Improvement in LDL and total cholesterol in the 9 month follow-up of our retrospective prolactinoma cohort as well as in the cohort presented here emphasize our previously published hypothesis that these parameters might be the first metabolic parameters affected by initiation of dopamine agonist treatment and that only continued use might lead to amelioration of further metabolic parameters.

Mechanisms of the now well documented association of hyperprolactinaemia and impaired lipid metabolism include the direct effect on adipose tissue and hypogonadism, as discussed above. After a median of only 10 weeks of cabergoline treatment, we already found a significant increase in total and free testosterone in men, but not in estradiol in women. In contrast, in our retrospective analysis we had indeed observed a significant increase in estradiol as well, suggesting that 10 weeks of treatment might be too short for an improvement in hypogonadism in all patients, or the number of women with available follow-up might have been too small to see a significant effect. Interestingly, in women alone, the decrease in total and LDL cholesterol was not significant, whereas it was indeed in men. Even though estradiol and total as well as free testosterone could not be identified as predictors of the change in total and LDL cholesterol in our retrospective cohort, our current findings presented here do suggest a role of hypogonadism in the development of adverse lipid parameters in patients with prolactinoma.

In contrast, as body weight was stable at follow-up, changes in lipid parameters seem to be independent of body composition [24] and might, in addition to hypogonadism, rather be a direct consequence of prolactin normalization or dopamine agonist-induced D2R activation, as previously suggested [2, 6]. Likewise, decreases in total and LDL cholesterol also preceded weight changes after initiation of cabergoline treatment in the biggest cohort published to date [10].

A number of studies have reported increased insulin resistance and reduced insulin sensitivity in patients with hyperprolactinaemia, supported by in vitro studies in the field [25]. However, as in our retrospective data, again we could not find evidence of overt impairment of glucose metabolism in our prolactinoma patients. Reasons for lacking effects on glucose metabolism might be the short duration of observation and the good glucose profile to begin with, demonstrated by the low number of patients with diabetes mellitus.

Whether altered lipid metabolism is detrimental in terms of cardiovascular risk and might increase mortality in patients with prolactinoma remains unanswered to date. In patients without prolactinoma, studies have yielded inconsistent results regarding this topic. In the Study of Health in Pomerania, an independent positive association of prolactin concentrations with all-cause and cardiovascular mortality in 3929 patients was reported [26]. In 3232 individuals from the Framingham Heart Study prolactin was not associated with a comprehensive panel of incident cardiovascular disease risk factors [27].

Certain limitations of our data have to be acknowledged including the design as a case-control study with a retrospective analysis of follow-up data. In addition, the number of patients analyzed is comparatively small and our data are prone to statistical type I errors due to multiple testing without correction. Our statistical analysis approach is, however, supported by the fact that our analysis plan was a priori designed and that all analyses were hypothesis driven. Furthermore, we cannot rule out a potential selection bias in the healthy control group, possibly explaining the differences in BMI between cases and controls and consequently the observed differences in lipid metabolism. These, however, remained unaltered after adjustment for BMI.

The main strengths are age-matched controls and the standardized treatment and follow-up of all patients. The significant impact of cabergoline treatment on prolactin and testosterone levels underlines the validity of our data.

## Conclusions

In conclusion, we observed higher levels of LDL cholesterol as compared to controls at baseline and a decrease of both total as well as LDL cholesterol after a short-term treatment with cabergoline in previously treatment-naïve patients. In addition, we found evidence of impaired body composition in terms of higher BMI in all patients and in terms of higher body fat mass in men with prolactinoma compared to controls. This increased metabolic risk – most likely triggered by hypogonadism – highlights the necessity to monitor lipid metabolism in patients with prolactinoma and sets the implication for future studies to elucidate the underlying pathophysiological mechanisms for the supposed link between prolactin and lipid metabolism. Adequate control of hyperprolactinaemia should be sought in order to reduce the possible risk of metabolic complications in patients with prolactinomas.

## Data Availability

The datasets generated and/or analysed during the current study are not publicly available due them containing information that could compromise research participant privacy/consent but are available from the corresponding author on reasonable request.

## Abbreviations

BMI: Body mass index
HOMA-IR: Homeostasis model assessment insulin resistance
HbA1c: Glycated haemoglobin A1c
LDL: Low-density lipoprotein
HDL: High-density lipoprotein
MRI: Magnetic resonance imaging
PCSK9 inhibitors: Proprotein convertase subtilisin/kexin type 9
TT: Total testosterone
FT: Free testosterone
fT3: Free triiodothyronine
fT4: Free thyroxine
TSH: Thyroid-stimulating hormone
ACTH: Adrenocorticotropic hormone
HGH: Human growth hormone
IGF-1: Insulin-like growth factor 1
SD: Standard deviation
IQR: Interquartile ranges
LH: Luteinizing hormone
FSH: Follicle-stimulating hormone
CRP: C-reactive protein
